# Survival was Significantly Better with Surgical/Medical/Radiation Co-interventions in a Single-Institution Practice Audit of Frameless Stereotactic Radiosurgery

**DOI:** 10.7759/cureus.612

**Published:** 2016-05-17

**Authors:** Amandeep Taggar, Joanna MacKenzie, Haocheng Li, Harold Lau, Gerald Lim, Robert Nordal, Alana Hudson, Rao Khan, David Spencer, Jon-Paul Voroney

**Affiliations:** 1 Oncology, University of Calgary; 2 NHS Lothian; 3 Statistics, University of Calgary; 4 Radiation Oncology, Tom Baker Cancer Centre, Calgary; 5 Medical Physics, Tom Baker Cancer Centre, Calgary; 6 Radiation Oncology, Washington University School of Medicine; 7 Oncology, Foothills Medical Centre

**Keywords:** Stereotactic Radiosurgery, brain metastases, neurosurgery, systemic therapy, whole brain radiotherapy

## Abstract

**Purpose:**

To audit outcomes after introducing frameless stereotactic radiosurgery (SRS) for brain metastases, including co-interventions: neurosurgery, systemic therapy, and whole brain radiotherapy (WBRT). We report median overall survival (MS), local failure, and distant brain failure. We hypothesized patients treated with SRS would have clinically meaningful improved MS compared with historic institutional values. We further hypothesized that patients treated with co-interventions would have clinically meaningful improved MS compared with patients treated with SRS alone.

**Methods:**

One hundred twenty patients (N = 120) with limited intracranial disease underwent 130 frameless SRS sessions from April 2010 to May 2013. Median follow-up was 11 months. MS was measured from brain metastases diagnosis, local failure, and distant brain failure from the time of first SRS.

**Results:**

Practice pattern during the first year of the study favored upfront WBRT (79%) over SRS (21%) while upfront SRS (45%) was almost as common as upfront WBRT (55%) in the last year of the study. MS was 18 months; 37% received SRS alone as initial radiotherapy (MS 12 months); 63% received WBRT prior to SRS (MS 19 months); 50% received systemic therapy post-SRS (MS 21 months); and 26% had tumor resection then SRS to the surgical cavity (MS 42 months). Local failure occurred in 10% of lesions and radio-necrosis occurred in 4%. Differences in distant brain failure among patients treated with upfront SRS (40% rate), WBRT followed by SRS (33% rate) or systemic therapy post-SRS (37% rate) were not statistically significant.

**Conclusion:**

Frameless SRS effectively treats surgical cavities, persistent tumors post-WBRT, and can be used as an upfront treatment of brain metastases. Surgery, systemic therapy, and WBRT are associated with longer MS. Patients can live for years while receiving multiple therapies. Systemic therapy for patients with brain metastases is increasingly common, palliative care occurs earlier and improves survival, and WBRT use is not routine. Modern series sometimes produce unexpectedly good results. Classification and treatment protocols are evolving. This practice audit is note-worthy for (i) high median overall survival, (ii) systemic therapy after radiosurgery for patients with tumors treated by radiosurgery, (iii) distant brain failure not significantly related to WBRT, and (iv) neurosurgery, systemic therapy, and WBRT are independently associated with improved MS.

## Introduction

Brain metastases cause significant morbidity and mortality [1]. With uncontrolled intracranial disease, patients often are not eligible for clinical trials. Whole brain radiotherapy (WBRT) and/or supportive care [2] are standard for patients with multiple brain metastases. Surgical and radiotherapy interventions can control limited intracranial disease [1-4]. Surgical resection is considered for patients with solitary lesions and good performance status, or for bleeding, edema or tissue diagnosis [3]. Stereotactic radiosurgery (SRS) can achieve local control with or without surgery or WBRT [5-7]. SRS/WBRT/supportive care are options at intracranial progression.

SRS can be delivered with a headframe pinned to the skull or with a frameless technique using an aquaplast mask or bite-block for immobilization. Frameless SRS is noninvasive and comfortable. It allows for multiple treatments, improved workflow and lower cost [8-9]. Concerns with frameless SRS are higher local failure or higher toxicity; emerging studies and our data refute this [10-13].

We report a contemporary sequential cohort of 120 patients treated with frameless SRS; some patients also received neurosurgery, systemic therapy, and WBRT. Median overall survival (MS) and co-intervention rates were primary endpoints; rates of local failure, distant brain failure, and radio-necrosis were secondary endpoints. We postulated that patients with frameless radiosurgery have a clinically important MS improvement of three months over our institution's historic (2000-2005) MS of 3.1 months [14], and those receiving multi-modality treatment have further three or more months improvement in MS over those treated with frameless SRS alone.

## Materials and methods

Consecutive patients from a Canadian catchment area (5 million people living in a two million km^2^ area) were treated at the catchment’s single radiosurgery center. Patients were treated in compliance with a protocol approved by the Alberta Cancer Research Ethics Review Committee, and constituted our initial experience with frameless SRS. Patients were offered radiosurgery after multidisciplinary review, including neurosurgery, radiation oncology, medical physics, and nursing. Eligibility per session was restricted to four or fewer brain metastases each under 3.5 cm. One patient had five metastases treated in two sessions separated by one week. We conducted clinical follow-up and MRI routinely every three months. We treated two patients with primary brain tumors (ependymoma and acoustic neuroma), who were excluded from analysis. Two patients from Saskatchewan had demographic information but no follow-up, and were censored at the first event. We prospectively recorded performance status, histology, systemic therapy, surgical resection of brain metastases, and prior WBRT. Patients with indications were referred to palliative care, nutrition, occupational and physiotherapy, home care and spiritual care [15]. Driving advice, seizure information, personal directive assistance, and goals of care advice were routinely given. Patient, disease, and treatment characteristics are presented in Table 1.

**Table 1:** Patient, disease, and treatment characteristics. KPS: Karnofsky performance status; RPA: recursive partitioning analysis; PTV: planning target volume (tumor plus a 1 mm margin, 2 mm for surgical cavities); WBRT: whole-brain radiotherapy; GI: gastro-intestinal; GU: genito-urinary; HNC: head and neck carcinoma. *univariable analysis for survival, p-value; †multivariable Cox proportional-hazards model for survival, p-value.

		uni^*^	multi^†^
Total number of patients	120		
Total number of SRS sessions	130		
Total number of lesions treated	239		
Age, years		0.138	0.540
Median (Range)	59.6 (26.3 – 80.7)		
Gender		0.369	0.592
Male	52		
Female	68		
KPS		0.002	0.702
100	7		
90	44		
80	38		
70	26		
<70	5		
RPA		<0.001	
1	24		
2	90		0.329
3	5		0.034
Lesions per patient, Median (Range)	1 (1-4)		
Histology			
Lung	53		
Breast	21		
Melanoma	20		
Renal Cell Carcinoma	10		
GI (Colon & Rectal)	7		
GU/Gyne	5		
Other	4		
Treatment, number of patients			
SRS alone	48		
WBRT followed by SRS	74	0.004	0.014
Chemotherapy post SRS	58	0.05	0.012
Surgery prior to SRS	31	<0.001	<0.001
SRS Radiation dose to 80% isodose, cGy			
Median (Range)	1800 (1200-2400)		
WBRT dose, cGy			
Mean (Range)	2800 (2000-3750)		
WBRT number of fractions			
Mean (Range)	9 (9-15)		

### Treatment set up and planning

Patients had computed tomography (CT) simulation with a clamshell thermoplastic mask. Gross tumor volume was outlined using the diagnostic magnetic resonance imaging (MRI) fused with the planning enhanced CT scan on the Brainlab^TM^ or Iplan^TM^ system (Brainlab Inc., IL, USA). Scans had 1 mm slice thickness. Tumor volume to planned treatment volume expansion margin was a symmetric 1 mm. Treatment was delivered on a Novalis^TM^ linear accelerator (Brainlab Inc., IL, USA) using 6 MV photons with either cones or micro-multileaf collimators and dynamic conformal arcs. Patients were set up using a target positioning system box, where the isocenter was marked on the box in three planes and treatment unit lasers used to align the marked isocenter with the unit’s isocenter. The location of the unit’s isocenter was calibrated daily with a Winston-Lutz test. Position was verified by matching dynamic digitally reconstructed radiographs of the planning CT with patient images obtained from an ExacTrac^TM^ image guidance system. Tolerance was 0.6 mm for translational deviations and 2^0^ for angular deviations. Patients were imaged with ExacTrac^TM^ (Brainlab Inc., IL, USA) between arcs, and repositioned if tolerances were not met. Accuracy and reproducibility of this technique have been validated at our centre (unpublished abstract data available from the authors, Banff Alberta Cancer Foundation Conference, Taggar/Voroney, 2010) and others [10].

### Analysis

Diagnosis of local failure is clinical and radiologic, often without pathology. Features of local failure include: increasing lesion gadolinium enhancement on consecutive MRIs; enhancement outside the 12 Gy isodose volume; lack of reduction in enhancement despite four weeks of steroid therapy; increase in maximum diameter of enhancement of 25% or 5 mm. It is often not possible to determine local failure versus radionecrosis with a single scan, and follow-up MRI at four to six weeks is often required to confirm the initial diagnostic impression. Distant brain failure was defined as a new lesion 2 cm or more from sites of previous SRS. Radionecrosis was defined as increased enhancement that, with or without steroids, did not progress on subsequent post-treatment MRIs. Patients were recorded as having surgery if they received surgery for brain metastases at any time prior to SRS; no patient received neurosurgery or autopsy post SRS.

We determined patients’ extracranial disease status by clinical or imaging evidence of extracranial disease at their most recent follow-up prior to SRS. The initial assessment was used to determine recursive partitioning analysis (RPA) score [12].

Kaplan-Meier curves were used to estimate MS. Univariate logistic regression was performed to test association of MS with prognostic variables (RPA) and co-intervention variables (WBRT, neurosurgery, systemic therapy post SRS). The variables were fitted into a Cox-regression model for multivariate analysis. All analyses were done using SPSS (version 22) (IBM Corporation, NY, USA) and graphs were generated on MedCalc (version 14.10.2) statistical software (MedCalc Software bvba, Ostend, Belgium).

## Results

Table 1 lists patient characteristics, and disease and treatment factors: 231 metastatic lesions in 120 patients were treated in 130 frameless SRS sessions. One patient with five lesions had treatment delivered on two separate days a week apart for patient convenience and was counted as a single session. The median number of target lesions treated in a single session was one (range, R 1–4). Two lesions within 1 cm were treated with a single isocenter and were counted as a single lesion. The median prescription dose was 18 Gy (R 12–24 Gy) prescribed to the 80% isodose line. Lower dose (12-14 Gy) was used near brainstem or optic chiasm. 100% of the planning target volume received ≥ 99% of the prescription dose. Patients with at least one follow-up or death were included in the analysis (117 patients with 229 lesions). Median follow-up from the time of brain metastasis diagnosis was 11 months (R 1.4–60) and 5.9 months (R 0–29) from time of SRS. Uncommon failure is reported in Table 2.

**Table 2:** Local and regional control rates

Local and Regional Control Rates	
Local failure, number (% of lesions)	22 (10%)
Mean time to local failure (95% CI), months	6.8 (5.5 – 7.7)
Distant brain failure, number (% of patients)	45 (38%)
Mean time to distant brain failure (95% CI), months	4.5 (3.4 – 5.7)

### Survival

**Figure 1:**
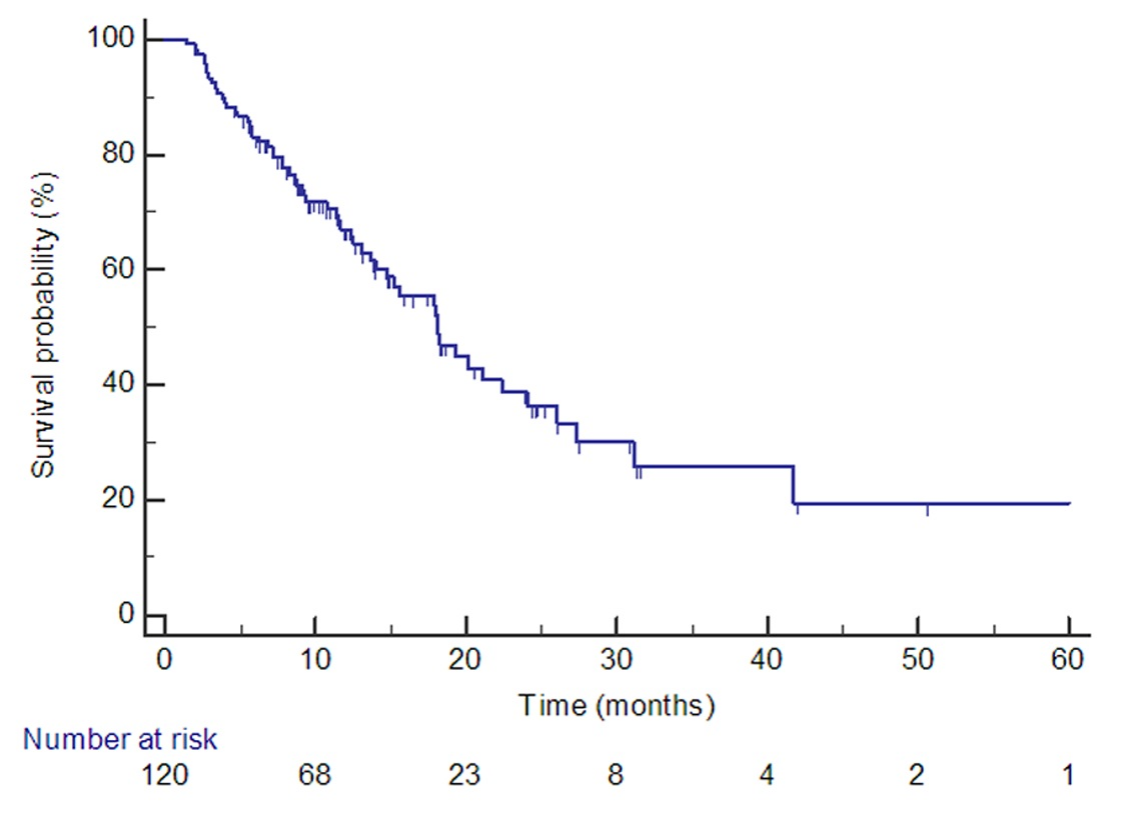
Survival probability of patients treated with frame-less SRS. MS = 18 months (95% CI 14–22). 75% survive to 10 months, 40% survive to 20 months and 30% survive to 30 months or more. SRS: stereotactic radiosurgery; CI: confidence interval; MS: median overall survival.

MS from time of brain metastasis diagnosis was 18 months (95% confidence interval (CI) 14–22) (Figure 1).

**Figure 2:**
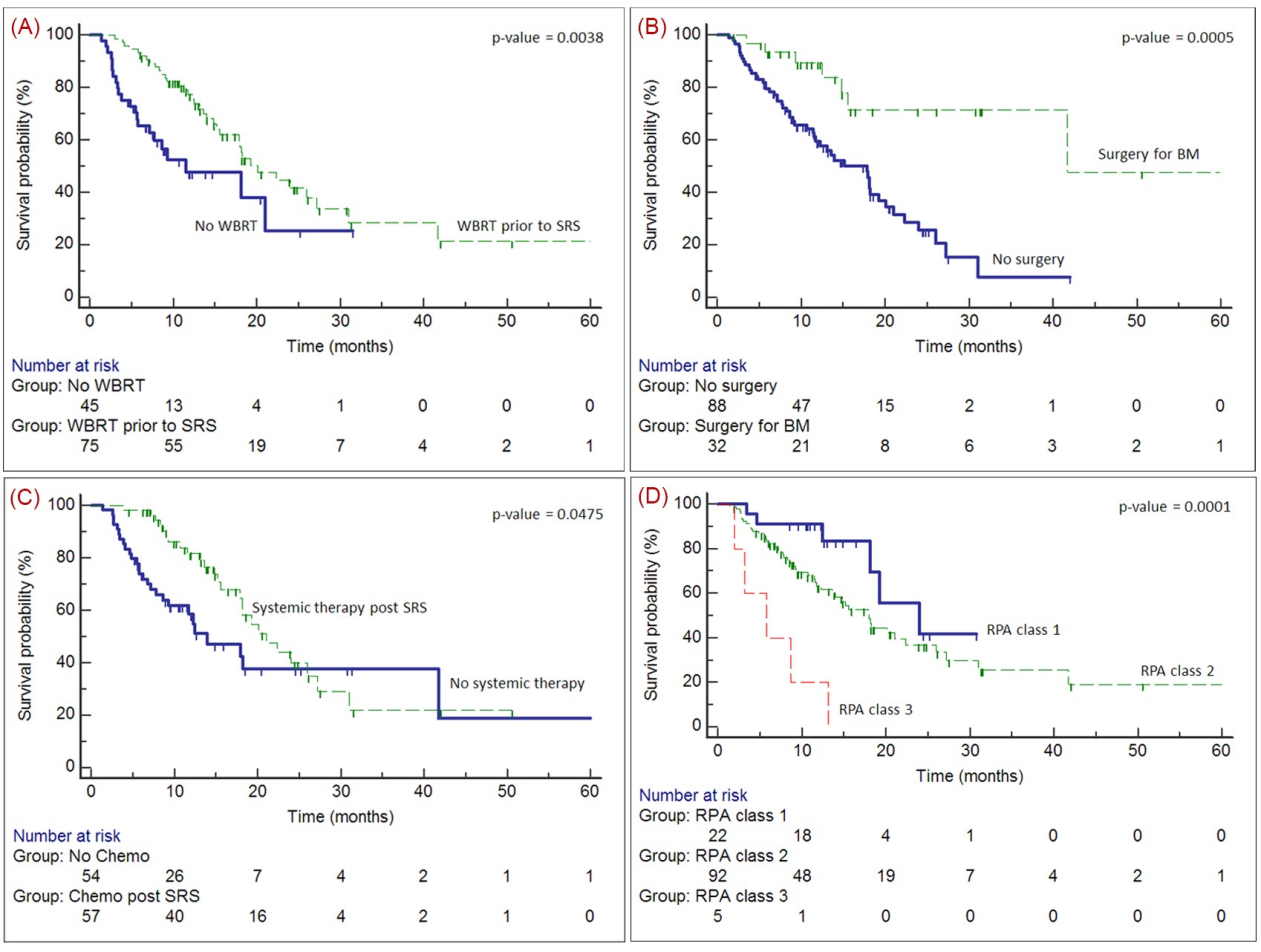
Survival probability from time of brain metastasis diagnosis: WBRT prior to SRS (A); surgery prior to SRS (B); systemic therapy post-SRS (C); and RPA (D). WBRT: whole-brain radiotherapy; systemic therapy: systemic therapy; RPA: recursive partitioning analysis; CI: confidence interval; MS: median overall survival.

Actuarial survival rates at six, 12, and 24 months were 83%, 67%, and 39% respectively. MS of 32 patients who had surgical resection prior to SRS was 42 months (95% CI 16–67), compared to 18 months (95% CI 14–22) for those who did not (P<0.001) (Figure 2).

MS of 78 (65%) patients who received WBRT prior to SRS was 20 months (95% CI 15–25) compared to 12 months (95% CI 2.5–21) for 42 patients whose initial treatment was SRS alone (P=0.004), making interpretation difficult. Half of the patients were subsequently treated with systemic agents post SRS (57/118). For patients with systemic therapy after SRS, MS was 21 months (95% CI 16–26) compared to 14 months (95% CI 7.7–20) for patients who had no systemic therapy post-SRS (P=0.05).

When stratified by RPA, MS of patients with RPA classes 1 to 3 were 24 months (95% CI 12–36), 18 months (95% CI 14–22) and 5.8 months (95% CI 0.1–12) respectively, (P<0.001), (Figure 2d). Actuarial 12-month survival according to RPA classification was 91%, 64%, and 20% for classes 1, 2, and 3, respectively. Patients in RPA class 2 did significantly better if they received systemic therapy post SRS (MS = 22 vs 12 months, P=0.02). MS for RPA class 2 patients who received systemic therapy and RPA class 1 patients were similar (22 vs 24 months P=NS), suggesting that patients with no systemic disease did as well as patients with systemic disease who were eligible for systemic therapy.

### Local failure in the treated lesion and distant brain failure

Table 2 summarizes the local failure and distant brain failure rates. We observed a local failure rate of 10% (22/229 lesions). Median time to local failure was 5.9 months (R 1.0–18.0). In a multivariable model patient age, KPS, surgery or WBRT use prior to SRS and systemic therapy post SRS were *not* found not to be associated with rates of local failure. Distant brain failure was *not* associated with WBRT, and median time to distant brain failure was 3.3 months (R 1.3–23), when it occurred. Nine patients had repeat SRS for new intracranial lesions. This includes two who had radiosurgery three times. One patient had whole brain radiotherapy followed by further radiosurgery.

### Toxicity

Using the RTOG acute radiation morbidity scoring criteria (CTCAE 4.0), one grade ≥3 toxicity was observed, for a patient who seized the evening after radiosurgery. The patient was on an anticonvulsant due to prior seizure and refused steroids for treatment of intracranial edema, due to prior debilitating steroid-psychosis. The patient fell and fractured a T-spine, requiring vertebroplasty.

Radio-necrosis was the most common late toxicity and was radiologically identified in four percent of treated lesions. Progressive disease versus radionecrosis often requires serial imaging to determine likely etiology: tumor or iatrogenic. One patient needed corticosteroids for symptomatic radionecrosis. Neurosurgery post-radiosurgery for radionecrosis was not needed in this series.

## Discussion

This frameless SRS cohort occurs during increases in multidisciplinary care, including palliative care and systemic therapy, at our institution [15]. Multidisciplinary care can change practice: for example, futile radiotherapy at our institution, defined as SRS or WBRT followed by death within four weeks is decreasing [14-15]. Two frameless SRS patients died within four weeks of treatment. Our SRS population data, supports a 40% survival at two years after brain metastasis diagnosis.

Radiosurgery is increasing at our institution: 27% per year over the last three years. As extracranial disease is better controlled with systemic therapy, it is increasingly important to control intracranial disease and prevent neurologic death. We identified patients who benefit from interventions such as neurosurgery, SRS, and WBRT. SRS is established as standard treatment for patients with ≤4 intracranial lesions, as sole initial treatment, or combined with WBRT, or as salvage after WBRT. Less established is SRS for surgical cavities, as in the NCCTG N107C protocol. Technological advances have made it possible to deliver high doses of radiation to complex intracranial targets. Achieving accurate localization has enabled development of frameless SRS: more comfortable for patients and deliverable on a flexible schedule. There is no need for a patient to wait in the department with a head-frame, while the equipment is repaired or a physician deals with a crisis. The frameless patient can go home and be treated the next day or next week.

We report outcomes using linac-based, BrainLab ExacTrac^TM^ kV imaging-directed SRS (Brainlab Inc., IL, USA). Patients are reliably immobilized using a thermoplastic mask [16]. Bony anatomy, matched with kV imaging, precisely locates intracranial targets. High local control and low complication rates are comparable to results published in framebased and frameless series [10-13], [17-20]. Quality of life was not formally evaluated; however patients treated with frameless technique avoid a day with a rigid head-frame pinned to their skull. Neurosurgery, radiation oncology, and medical oncology are extensively involved in our radiosurgery population.

We report median overall survival of 18 months as compared to 8-15 months reported in the SRS literature [10-13], [17-20]. The improved MS may reflect lead-time bias as 62% of the patients had WBRT prior to SRS, and MS was measured from first radiographic evidence of brain metastases. Nonetheless, this MS is clinically important to our patients, and improved over patients treated with supportive care or WBRT only in the past. [14-15, 21]. A systematic review of randomized controlled trials evaluating WBRT alone versus WBRT plus SRS showed that patients with solitary lesions and SRS have a significant improvement in local control that may translate into improvement in MS [1]. We observed that patients who received WBRT prior to SRS had significantly longer survival (P=0.004). This is confounded by offering SRS alone to patients who do not meet previously used criteria, in order to avoid side effects of WBRT, resulting in *decreased* survival for SRS alone patients due to relaxed patient selection. Nevertheless, MS for patients treated with SRS alone was 12 months, comparing well with other series.

Patchell et al. first reported control rates of 89% in patients treated with surgery plus WBRT for single intracranial metastases [3]. This important trial occurred 25 years ago. With modern imaging and radiosurgery planning techniques, local failure rates with frame-based SRS range from 10-20% [22-24] and from 2-20% [10,13], [17-20] with frameless SRS. Low local failure was achieved in this cohort: frameless SRS is effective treatment for metastatic brain lesions. Furthermore, 32 patients had surgical resection with SRS following surgery. MS of these patients was 42 months and local failure rate was 16%, similar to or improved over previously reported outcomes of postoperative SRS to surgical beds [20,25,26].

We examined the influence of systemic treatments (chemotherapy or immunotherapy) on MS, local failure, and distant brain failure rates. Despite paucity of literature on this topic, limited available data suggests systemic therapy post SRS may improve both local control and MS [27-28]. Furthermore, limited data exists on timing of SRS in the context of uncontrolled systemic disease; whether treatment of asymptomatic intracranial disease prior to systemic therapy improves MS or local control has not been established. A best practice study would state upfront meaningful benefits including quality of life, would randomize, and would stratify according to histology and available treatments. A phase 3 Radiation Therapy Trials Group study randomized 126 patients to WBRT and SRS +/- temozolomide or erlotinib for metastatic non-small cell lung cancer (NSCLC) with 1-3 cerebral metastases. This study showed no improvement in time to distant brain failure/local failure/MS rates with the addition of systemic therapy post SRS [29]. Muacevic et al. reported 60% of 333 patients had systemic therapy use in patients treated with SRS for brain metastases [20]. The timing of systemic therapy and SRS was not stated and differences in MS were not reported. Mori et al. [28] examined factors that affected MS and local failure in 60 patients treated with SRS for metastatic melanoma. Systemic therapy post SRS improved MS and local failure in univariable analysis, but not in multivariable analysis [28]. We observed that systemic therapy post SRS was associated with improved MS (P=0.05) that remained significant in a multivariable model (P=0.01), particularly for patients younger than 60 years and/or those with RPA class of 2. Systemic therapy use was not significantly associated with rates of local failure or distant brain failure (P=0.51 and P=0.81, respectively), suggesting the need to follow SRS patients closely.

### Limitations

This study has inherent biases: lack of randomization or a cohort of well-described controls, heterogeneous patient population, diverse histology, and various salvage systemic treatments, which confound interpretation of SRS and co-interventions. Nevertheless, our results suggest patient selection may significantly improve outcomes, and patients eligible for multi-modality treatment may have superior survival.

## Conclusions

This practice audit of frameless radiosurgery and co-interventions shows improved results of MS of 18 months compared to our institution’s MS of 3.1 months for 555 patients with brain metastases treated between 2000 and 2005 [14], prior to changes in multi-disciplinary and radiosurgery management. Quality of life is important when treating patients with brain metastases: some patients achieve durable disease control; few patients survive beyond four years from a diagnosis of brain metastases. Frameless SRS is well tolerated. Further treatment may involve neurosurgery, systemic therapy, palliative care, and/or whole brain radiotherapy.

This large, contemporary series validates effectiveness of frameless SRS for ≤4 intracranial metastases with low local failure rate and low toxicity, for our institution’s population. This technique offers SRS for brain metastases without neurosurgical head-frame placement. Neurosurgery, especially for single lesions, remains an integral part of frameless radiosurgery, in particular the treatment decision-making and consultation processes. Medical oncologists increasingly treat select patients post radiosurgery with systemic therapies, and given the evidence for systemic therapies, our results reflect the importance of follow-up, intracranial control, and where appropriate, palliative care and medical oncology involvement.
